# Eye and Vision (E & V): the critical link between eye and vision

**DOI:** 10.1186/s40662-014-0001-3

**Published:** 2014-10-16

**Authors:** Jia Qu

**Affiliations:** Editor-in-Chief, Eye and Vision, Wenzhou Medical University, Wenzhou, China

“The eye is the window to the soul” – this age-old adage succinctly encapsulates the vital importance of the complex and organic structure of the eye and its involvement in the functionality of vision and perception. In most instances however, the eye and vision tend to be addressed as separate entities and the interrelation between form and function then gets neglected. As a result, we fail to acknowledge or recognize that this critical gap between the eye and vision hold the key to more efficient and effective diagnosis and treatment of common eye diseases and vision problems like diabetic retinopathy, age-related macular degeneration, cataract, and glaucoma.

In the past decades, we have witnessed dramatic increases in prevalence and incidence of eye diseases and vision impairment and these have garnered increased attention in the medical and scientific fields because they pose significant public health concerns. According to the WHO (2013), an estimated 285 million people are visually impaired globally. Major causes of visual impairment worldwide include uncorrected refractive errors (43%), unoperated cataract (22%), and glaucoma (2%) www.who.int/mediacentre/factsheets/fs282/en/. Furthermore, it is expected that by the year 2020, approximately 37 million worldwide would be blind or have low vision. The consequences then, of our oversight regarding the gap between the eye and vision has led to increased economic burden to the society and psychosocial problems like depression, fear and anxiety, unemployment, and divorce.

With the graying of the worldwide population that is responsible for a large part of these seemingly catastrophic numbers, what can be done to avert this undesirable occurrence? These otherwise alarming statistics may be prevented if more effort was devoted to bridging the disciplines of ophthalmology, optometry, and the visual sciences. Since 80% of all visual impairment globally is said to be preventable or curable, we need to work toward encouraging collaborative research and the open sharing of knowledge between ophthalmologists, optometrists, visual scientists and others, in order to disseminate accurate, reliable, and applicable information with the hopes of crippling eye and vision diseases.

Therefore, *Eye and Vision* (*E* & *V*) being a peer-reviewed, international Open Access journal is devoted to the rapid publication of novel and exciting results on fundamental issues in all aspects of eye and vision that include (but is not limited to) current developments of theoretical, experimental and clinical investigations in ophthalmology, optometry and visual sciences, which focus on novel and high-impact findings on central issues pertaining to biology, pathophysiology and etiology of eye diseases as well as advances in diagnostic techniques, surgical treatments, instrument updates, and the latest research findings. The utmost goal of this journal is thus to provide a platform to bridge the inherent knowledge gap between studies of physical and structural aspects of the eye and its functionality in vision and perception.

